# WHAT IS IMPORTANT FOR THE FEELING OF MEANINGFULNESS? A STUDY OF SWEDES AGED 76–101 YEARS

**DOI:** 10.1093/geroni/igad104.2162

**Published:** 2023-12-21

**Authors:** Ingemar Kåreholt, Amelie Bergström Af Ekenstam

**Affiliations:** Jönköping University, School of Health and Welfare, Jönköping, Jonkopings Lan, Sweden; School of Health and Welfare, Jönköping, Jonkopings Lan, Sweden

## Abstract

Sense of coherence (SOC) reflects the coping capacity to deal with everyday life stressors and consists of comprehensibility, manageability and meaningfulness. Meaningfulness is the motivational component and sometimes considered the most important part in SOC. Meaningfulness is about the extent to which a person feels that life has an emotional meaning. Meaningfulness is important in aging since many old people suffer from health problems and meaningfulness is important for coping. We examine which factors that are important for the feeling of meaningfulness in a nationally representative random sample of 777 Swedes aged 76-101 years. Meaningfulness is measured with a single item question “Do you usually feel that your daily life is a source of personal satisfaction?”. 11% answered “no”, 20% “yes, sometimes”, 59% “yes, often”. Analyzed with ordered logistic regressions. Control variables: sex, age, education, frailty index, mobility, and psychological wellbeing. Mobility and psychological wellbeing were significant and kept in the analyses, The main independent variables were having a faith (or philosophy), cultural, intellectual, outdoor activities and the social variables loneliness, social contacts outside home, social relations to relatives and friends, contact with children/grandchildren, and phone contacts with children/grandchildren. The social variables were analyzed simultaneously. Loneliness, social relations to relatives and friends, phone contacts with children/grandchildren were significant as well as all other main independent variables. Analyzed simultaneously, controlled for each other, mobility and psychological wellbeing, faith, loneliness, social relations to relatives/friends, and contacts with children/grandchildren were significant. This indicates that these factors are important for meaningfulness in old age.

